# Burden of Early Mortality Attributable to Hodgkin Lymphoma in Young Adults: A United States Study Based on CDC WONDER Data (2018–2023)

**DOI:** 10.7759/cureus.106871

**Published:** 2026-04-12

**Authors:** Akoju Shiva Teja, Shibil Mohammad, Yuvaraj Korant, Unnimaya Mohandas

**Affiliations:** 1 Neurology, Citi Neuro Centre, Hyderabad, IND; 2 Internal Medicine, Family Health Centre, Palakkad, IND; 3 Medicine, Banas Medical College and Hospital, Palanpur, IND; 4 Internal Medicine, Divine Medical Mission Hospital, Vadakkencherry, IND

**Keywords:** cdc wonder, crude mortality rate, early-onset mortality, hodgkin lymphoma, retrospective study, young adults

## Abstract

Introduction

Hodgkin lymphoma is a malignant lymphoid neoplasm with a recognized predilection for adolescents and young adults. Although overall mortality is relatively low, deaths occurring in younger populations contribute substantially to years of life lost.

Aim

This study aimed to describe temporal trends and demographic patterns in mortality due to Hodgkin lymphoma among young adults aged 15-44 years in the United States between 2018 and 2023 using CDC WONDER (Centers for Disease Control and Prevention Wide-Ranging Online Data for Epidemiologic Research) data.

Methods

A retrospective population-based study was conducted using mortality data from the CDC WONDER database. Deaths between 2018 and 2023 with Hodgkin lymphoma as the underlying cause (ICD-10 code C81) were included. Analyses were restricted to individuals aged 15-44 years. Mortality counts, percentages, and crude mortality rates per 1,000,000 population were calculated and stratified by age, sex, and race/ethnicity.

Results

From 2018 to 2023, Hodgkin lymphoma accounted for 6,072 deaths across all age groups, of which 682 deaths (11.2%) occurred among individuals aged 15-44 years. Crude mortality rates in this population ranged from 0.8 to 0.9 per 1,000,000 over the study period, demonstrating relative stability. Mortality burden was highest among individuals aged 35-44 years, males, and non-Hispanic or Latino populations, indicating consistent demographic disparities.

Conclusions

Mortality due to Hodgkin lymphoma among young adults represents a measurable public health burden with persistent demographic disparities. These findings underscore the importance of early diagnosis, equitable access to care, and targeted public health strategies to reduce premature mortality in this population.

## Introduction

Hodgkin lymphoma (HL) is a malignant lymphoid neoplasm characterized by the presence of Reed-Sternberg cells within an inflammatory background. It mainly affects two age groups: adolescents and young adults [1]. In 2020, HL represented a meaningful share of cancer burden worldwide, with thousands of new diagnoses and hundreds to thousands of deaths, illustrating an ongoing mortality burden of 0.26 in 100,000 despite therapeutic advances [2]. HL incidence is highest in young adults aged 15-44 years, with a prominent peak in early adulthood reported by multiple epidemiological studies, making this age group particularly relevant for both clinical and public health planning [3]. Known demographic disparities in HL outcomes have been documented, including differences in incidence and survival by age, sex, and race/ethnicity [2].

The risk factors for HL are multifactorial and incompletely understood. Established risk factors include Epstein-Barr virus (EBV) infection, immunosuppression, and familial predisposition, all of which contribute to disease development in subsets of patients [1,4]. However, behavioral and biological factors relevant to young adults, such as immune system maturity, lifestyle factors, and patterns of healthcare access, have not been fully delineated in large cohorts. Evidence also suggests differences in disease presentation and aggressiveness by age group, with younger patients sometimes exhibiting distinct histological subtypes or symptomatic profiles compared with older adults [4]. Despite these observations, current explanations for early-onset mortality remain limited, as most risk models focus on incidence and long-term survival rather than early deaths within the first years after diagnosis.

Studying early deaths and demographic disparities in HL now is critical because premature mortality among young adults leads to substantial loss of potential years of life, amplifying the public health impact beyond absolute counts. Young adults who die early from HL face social and economic consequences, including reduced workforce participation and financial strain on families and caregivers [5]. Identifying demographic disparities in early-onset mortality is therefore essential for implementing proper health policies [5,6].

Early-onset mortality, defined as death due to HL occurring in individuals aged 15-44 years, represents a critical but underexplored outcome in young adults. Previous studies have consistently demonstrated excellent overall survival in young adults with HL; however, a subset of patients continues to experience early mortality.

Using large, nationally representative databases such as the CDC WONDER (Centers for Disease Control and Prevention Wide-Ranging Online Data for Epidemiologic Research) mortality database allows for precise trend analysis across age, sex, and race/ethnicity, enabling a focused assessment of mortality patterns that can inform future clinical and public health interventions.

This study aimed to describe temporal trends and demographic patterns (age, sex, and race/ethnicity) in mortality due to HL among individuals aged 15-44 years in the United States between 2018 and 2023, using data from the CDC WONDER database.

## Materials and methods

Study design and data source

A retrospective population-based observational study was conducted using mortality data from the CDC WONDER Multiple Cause of Death (MCD) database [7]. This database contains publicly available, deidentified mortality data derived from death certificates across the United States. Mortality data were extracted on December 16, 2025. In accordance with established guidelines for research using publicly available datasets, institutional ethics committee approval was not required, as this study did not involve human participants [8].

Study population

The study population included individuals aged 15-44 years who died between 2018 and 2023. Early-onset mortality was defined a priori as deaths occurring within this age range.

Outcome definition

The outcome of interest was mortality due to HL, defined as the underlying cause of death and identified using the International Classification of Diseases, 10th Revision (ICD-10) code C81.

Variables

Demographic variables included age group (15-24, 25-34, and 35-44 years), sex (male and female), and race/ethnicity (Hispanic or Latino and non-Hispanic or Latino). Records with missing or suppressed demographic data were handled in accordance with CDC WONDER's default reporting and suppression rules.

Mortality rate calculation

Mortality counts and crude mortality rates per 1,000,000 population were obtained directly from the CDC WONDER interface. Rates were standardized to the 2000 U.S. standard population as provided by the database [9].

Statistical analysis

All analyses were descriptive in nature. Mortality counts, percentages, crude mortality rates per 1,000,000 population, and corresponding 95% confidence intervals were obtained directly from the CDC WONDER platform using its built-in population-based calculation methods. No independent statistical software was used to perform additional inferential testing. Temporal trends from 2018 to 2023 were evaluated descriptively through year-to-year comparisons. Data organization was performed using Microsoft Excel MSO version 2511 (Microsoft Corporation, Redmond, Washington).

## Results

Overall mortality

From 2018 to 2023, mortality from HL across all age groups in the United States was n = 6,072, corresponding to a crude mortality rate of 3.1 per 1,000,000 population. However, among young adults aged 15-44 years, mortality due to HL was n = 682, with a crude mortality rate of 0.9 per 1,000,000 population. Mortality among young adults accounted for 11.23% of total HL-related mortality during the study period.

Characteristics of early-onset mortality

Table 1 shows that the highest proportion of deaths occurred in individuals aged 35-44 years, with 306 deaths (44.9%). This was followed by those aged 25-34 years, with 287 deaths (42.1%), and 15-24 years, with 89 deaths (13.0%). Regarding sex, deaths were more frequent among males, who accounted for 445 deaths (65.2%), compared with females, who accounted for 237 deaths (34.8%). For race/ethnicity, the majority of deaths occurred among individuals who were not Hispanic or Latino, with 560 deaths. This was followed by Hispanic or Latino individuals, with 120 deaths. Data for the "not stated" category were minimal and are reported as such in the table.

**Table 1:** Distribution of early-onset Hodgkin lymphoma mortality (aged 15–44 years) by age group, sex, and race/ethnicity—United States, 2018–2023 Values are given as numbers and percentages. NA - Not Available. Data suppressed (<10 deaths) as per CDC WONDER reporting rules.

Variables	Number	Percentage
Age, years		
15–24	89	13.0%
25–34	287	42.1%
35–44	306	44.9%
Sex		
Male	445	65.2%
Female	237	34.8%
Race		
Hispanic or Latino	120	NA
Not Hispanic or Latino	560	NA
Not stated	NA	NA

Overall, crude mortality rates among young adults aged 15-44 years remained largely stable over the study period, with minor year-to-year fluctuations. The study years shown include 2018 through 2023. The lowest crude mortality rate was observed in 2020 and 2021 (0.8 per million), while the highest rate was observed in 2018, 2019, and 2022 (0.9 per million). No sustained upward or downward trend was observed across the study period.

Age-specific crude mortality rates per 1,000,000 population varied across the study years. Individuals aged 35-44 years consistently demonstrated the highest crude mortality rates, ranging from 1.1 to 1.4 per million, with a peak observed in 2022. Those aged 25-34 years showed intermediate rates, ranging from 0.9 to 1.2 per million, with relatively stable values from 2020 to 2023. The 15-24-year age group demonstrated the lowest crude mortality rate, with a reported value of 0.5 per million in 2018, while data for subsequent years were unavailable (Table 2).

**Table 2:** Crude mortality rates (per 1,000,000 population) with 95% confidence intervals for early-onset mortality due to Hodgkin lymphoma by demographic characteristics—United States, 2018–2023 Values represent crude mortality rates per 1,000,000 population with corresponding 95% confidence intervals (rate: 95% CI).

Demographic characteristics	2018	2019	2020	2021	2022	2023
Overall	0.9 (0.8–1.1)	0.9 (0.7–1.1)	0.8 (0.6–1)	0.8 (0.7–1)	0.9 (0.7–1.1)	0.8 (0.7–1)
Age, years						
15–24	0.5 (0.3–0.7)	0.4 (0.2–0.6)	0.4 (0.2–0.6)	0.4 (0.2–0.6)	0.3 (0.1–0.5)	NA
25–34	1.2 (0.9–1.5)	1 (0.7–1.3)	0.9 (0.7–1.3)	1 (0.7–1.3)	1.1 (0.8–1.5)	1.1 (0.9–1.5)
35–44	1.1 (0.8–1.5)	1.3 (1.0–1.7)	1.1 (0.8–1.5)	1.1 (0.8–1.5)	1.4 (1.0–1.7)	1.1 (0.8–1.5)
Sex						
Female	0.7 (0.5–1)	0.6 (0.4–0.8)	0.7 (0.5–0.9)	0.5 (0.3–0.7)	0.7 (0.5–0.9)	0.5 (0.3–0.7)
Male	1.1 (0.9–1.4)	1.2 (0.9–1.4)	0.9 (0.7–1.2)	1.2 (1–1.5)	1.1 (0.9–1.4)	1.2 (0.9–1.5)
Race						
Hispanic or Latino	0.8 (0.5–1.2)	0.8 (0.5–1.2)	NA	0.9 (0.6–1.3)	NA	NA
Not Hispanic or Latino	0.9 (0.8–1.2)	0.9 (0.7–1.1)	0.8 (0.7–1)	0.8 (0.7–1)	1 (0.8–1.2)	0.9 (0.7–1.1)

Sex-specific crude mortality rates per 1,000,000 population showed consistently higher rates among males compared with females throughout the study period. Among males, crude mortality rates ranged from 0.9 to 1.2 per million, with minor fluctuations across years. Among females, rates were lower, ranging from 0.5 to 0.7 per million, with the lowest values observed in 2021 and 2023.

Race- and ethnicity-specific crude mortality rates per 1,000,000 population were highest among individuals who were not Hispanic or Latino, with rates ranging from 0.8 to 1.0 per million over the study period. Among Hispanic or Latino individuals, reported rates ranged from 0.8 to 0.9 per million in years with available data.

## Discussion

This study aimed to describe temporal trends and demographic disparities in early-onset mortality due to HL among adults aged <45 years in the United States between 2018 and 2023. The findings demonstrate a measurable burden of early-onset mortality within the overall HL mortality profile. Mortality was predominantly observed in the 25-44-year age group, with higher rates among males compared with females. This pattern may reflect differences in disease biology, healthcare access, or treatment response across demographic groups, as suggested in prior studies. Racial and ethnic differences were also evident, with higher mortality rates among non-Hispanic or Latino individuals compared with Hispanic or Latino individuals. Overall mortality trends during the study period showed relative stability with minor fluctuations, highlighting persistent demographic patterns rather than marked temporal shifts.

Early-onset or premature mortality refers to deaths occurring at younger ages than expected for a given condition and represents a significant public health concern due to the loss of productive life years and long-term societal impact [10,11]. Prior studies evaluating early or premature mortality in HL have used varying age thresholds, including <40, <45, and <50 years [12-14]. Despite differences in age definitions, these studies consistently report that a non-trivial proportion of HL-related deaths occur in younger populations.

The overall mortality trends observed in this study demonstrated relative stability across the study period, with minor year-to-year fluctuations. These findings are consistent with national registry-based studies reporting plateauing HL mortality despite therapeutic advances [15,16].

Age-stratified analyses revealed higher mortality rates among individuals aged 35-44 years compared with younger age groups. Similar age-related gradients have been reported in prior studies, despite differences in age-group categorization [12,14,17].

Sex-specific analyses demonstrated consistently higher mortality rates among males compared with females, aligning with prior epidemiological studies of HL [13,15,18].

Higher mortality rates were observed among non-Hispanic or Latino individuals compared with Hispanic or Latino individuals. Prior studies have similarly reported racial and ethnic disparities in HL outcomes, likely reflecting social and structural determinants of health [10,11,14].

Limitations

This study has several limitations. Death certificate-based data may be subject to misclassification or coding inaccuracies. The CDC WONDER database lacks individual-level clinical details (e.g., stage, treatment) and socioeconomic factors, limiting interpretation and introducing potential confounding. Suppression of small counts and possible misclassification of race/ethnicity may affect subgroup analyses. Finally, as a descriptive study using aggregated data, causal inferences cannot be established.

## Conclusions

This study demonstrates a meaningful burden of early-onset mortality associated with HL in the United States between 2018 and 2023. Mortality rates among young adults showed relative stability over time, with the highest burden consistently observed among individuals aged 35-44 years, males, and non-Hispanic or Latino populations, indicating clear demographic disparities. These findings underscore the importance of early diagnosis, risk-adapted surveillance, and equitable access to oncology care in younger adults. From a public health perspective, targeted awareness initiatives and improved healthcare engagement among high-risk demographic groups may help reduce premature deaths. Future research should incorporate clinical characteristics, treatment patterns, and socioeconomic determinants to better understand the drivers of early-onset mortality and to inform effective prevention and intervention strategies.
